# SG-APSIC1164: Cost-effectiveness of temporary isolation rooms in acute-care settings in Asia

**DOI:** 10.1017/ash.2023.85

**Published:** 2023-03-16

**Authors:** Nicholas Graves, Yiying Cai, Dale Fisher, Martin Kiernan, Brett Mitchell

**Affiliations:** Singapore General Hospital, Singapore; Singapore General Hospital, Singapore; Singapore National University Hospital, Singapore; University of West London, London, United Kingdom; Newcastle University, Newcastle, Australia

## Abstract

**Objectives:** We estimated the change to health-service costs and health benefits resulting from a decision to adopt temporary isolation rooms, which are effective at isolating the patient within a general ward environment. We assessed the cost-effectiveness of the decision to adopt temporary isolation rooms in a Singapore hospital. **Methods:** Existing data were used to update a model of the impact of adopting temporary isolation rooms on healthcare-associated infections. We predicted the expected change to health service costs and health benefits, measured in life years gained. Uncertainty was addressed using probabilistic sensitivity analysis, and the findings were tested with plausible scenarios to determine the effectiveness of the intervention. **Results:** We predicted 478 fewer HAIs per 100,000 occupied bed days resulting from a decision to adopt temporary isolation rooms. This decreased would result in cost savings of SGD$329,432 (US $247,302) and 1,754 life years gained. When the effectiveness of the intervention was set at 1% of cases of HAI prevented, the incremental cost per life year saved was SGD$16,519 (US $12,400), indicating that this would be a cost-effective measure in Singapore. **Conclusions:** We have provided evidence that adoption of a temporary isolation room would be cost-effective for Singapore acute-care hospitals. Using temporary isolation rooms may be a positive decision for other countries in the region with fewer resources for infection prevention and control.

